# Rapid and Reliable HPLC Method for the Simultaneous Determination of Dihydroxyacetone, Methylglyoxal and 5-Hydroxymethylfurfural in *Leptospermum* Honeys

**DOI:** 10.1371/journal.pone.0167006

**Published:** 2016-11-18

**Authors:** Matthew Pappalardo, Linda Pappalardo, Peter Brooks

**Affiliations:** School of Science and Engineering, University of the Sunshine Coast, Maroochydore, QLD, Australia; University of Colorado Denver School of Medicine, UNITED STATES

## Abstract

A reliable determination of dihydroxyacetone, methylglyoxal and 5-hydroxymethylfurfural is essential to establishing the commercial value and antimicrobial potential of honeys derived from the *Leptospermum* species endemic to Australia and New Zealand. We report a robust method for quantitation of all three compounds in a single HPLC run. Honey samples (n = 6) that are derivatized with *o*-(2,3,4,5,6-Pentafluorobenzyl) hydroxylamine were quantitated against a stable anisole internal standard. Linear regression analysis was performed using calibration standards for each compound (n = 6) and results indicated a high degree of accuracy (R^2^ = 0.999) for this method. The reliability of some commercial methylglyoxal solutions were found to be questionable. Effective quantitation of methylglyoxal content in honey is critical for researchers and industry, and the use of some commercial standards may bias data. Two accurate methylglyoxal standards are proposed, including a commercial standard and a derivative that can be prepared within the laboratory.

## Introduction

Honey produced from the nectar of *Leptospermum* species has captured interest because of its potential health benefits and antibacterial properties. Methylglyoxal (MGO) has been reported as the primary compound responsible for the non-peroxide antibacterial activity (NPA) of *Leptospermum scoparium* (mānuka) honey from New Zealand [1, 2]. MGO was found to accumulate in these honeys due to an inverse, time-dependent chemical conversion from 1,3-dihydroxyacetone (DHA) sourced from the nectar of *L*. *scoparium* [3]. These compounds have also been identified in Australian *Leptospermum spp*. honeys [4, 5] and there has been a substantial commercial value resulting from these findings for industry, as honeys with high MGO content are priced at a premium.

The concentration of MGO in honey was initially determined by a direct method utilizing High Performance Liquid Chromatography (HPLC) linked with Refractive Index monitoring, although this method had low sensitivity [1]. Consequent analyses improved quantitation by prior derivatization of MGO with *o*-phenylenediamine, however this protocol is time inefficient [1, 6].

The first efficient method for simultaneous quantitation of MGO and DHA utilized derivatization with *o*-(2,3,4,5,6-Pentafluorobenzyl) hydroxylamine (PFBHA) to tag the compounds for analysis by HPLC linked to a Photodiode Array Detector [4]. This method was adapted from a quality control protocol for DHA in tanning creams, where the compound is an active ingredient [7]. This timely and accurate technique for analysis has been adopted by industry and in research [8–10].

The PFBHA method provided rapid quantitation by incorporating an internal standard hydroxyacetone (HA) and was later optimized to quantitate 5-hydroxymethylfurfural (HMF), a marker associated with honey spoilage or heat treatment [8, 11]. As conversion of DHA to MGO occurs over time, it is critical to monitor the HMF content within these honeys to ensure their safety while maximizing their commercial viability.

Although the PFBHA method has distinct advantages, our laboratory has observed problems associated with reproducibility and efficiency. The HA internal standard degrades with storage and is therefore limited to a single use only. In addition, the concentration of some commercial MGO standards and solutions have varied from their purported concentrations, interfering with calibrations and quantitation. As the commercial value of honeys are often directly influenced by MGO content, it is critical to ensure accountable and consistent quantitation of this compound. Therefore, we aimed to improve the productivity and repeatability of the PFBHA method and also necessary to clarify the reliability of MGO standards.

## Materials and Methods

### Chemicals

HPLC Chromasolv grade acetonitrile (ACN) (Merck, Kilsyth, Australia) was the primary solvent for HPLC analysis and water for use in this method was obtained from in-house MilliQ^®^ system. Two MGO solutions with approximately 40% w/w, Anisole 99%, HMF 99.1% and DHA 99% were purchased from Sigma Aldrich (Ryde, Australia).

A 1001 ppm AccuStandards MGO standard was purchased from Phenomemex (Lane Cove, Australia) for use as a MGO calibration standard. In addition, a commercial MGO standard (name withheld, 1003 ppm w/w) was obtained for comparison of MGO standards.

PFBHA hydrochloride was purchased from Alfa Aesar (Lancashire, United Kingdom) and reagent grade citric acid and sodium hydroxide were purchased from Chem Supply (Port Adelaide, Australia).

### Solvents, Reagents and Standards

The derivatization reagent was prepared by dissolving PFBHA hydrochloride at 200 mg/15.0 mL in a 0.1 M citric acid buffer solution, adjusted to pH 4.0 with 1 M NaOH.

A bis-derivative MGO was prepared by mixing 100 μL of 40% w/w MGO solution with 20 mL PFBHA derivatization reagent (200 mg/10 mL citric acid buffer) in a calibrated molar ratio (MGO: PFBHA 1:2.5). After dilution with an equal volume of ACN (20 mL) the solution was stirred (30 minutes) at 40–50°C, then cooled over ice. The precipitant was recovered by vacuum filtration, re-dissolved in 5 mL of hot ACN and then slowly cooled to allow recrystallization. The crystals are washed in cold ACN: water (50:50, v/v) and desiccated. The MGO-bisPFBHA standard was prepared from this recrystallized derivative (89.0 mg/ 20.0 mL ACN).

HMF and DHA were prepared fresh as a dual standard by combining 64.3 mg and 15.6 mg of each compound respectively in 50.0 mL water. Anisole was used as an internal standard and it was made by adding 300 μL of Anisole to one liter of dilution solvent (water: ACN 25/75, v/v).

### Calibration Standards

Approximately 300 mg of a honey previously determined to contain no DHA or MGO was weighed into six tared 16 x 75 mm glass tube and these masses were recorded. Aliquots of the MGO-bisPFBHA standard dissolved in ACN (0, 50, 100, 200, 400 and 800 μL) were added by pipette and the mass of each aliquot was also recorded to four decimal places. The honey and standard solutions were mixed with derivatizing reagent (2.0 mL) by vortex and then by glass Pasteur pipette, which was then left in the solutions during derivatization at room temperature.

After 75 minutes, the tubes were tared and 6.0 mL of dilution solvent with internal anisole standard was added by 10.0 mL pipette. This mass was recorded before thorough mixing. Extra ACN (100%) was added to samples where the MGO derivative had not fully dissolved. Aliquots (~1.0 mL) were analyzed by HPLC.

The dual DHA and HMF standard was added in 0, 50, 100, 200, 500 and 1000 μL aliquots to tared glass test tubes that did not contain honey and the masses were recorded to four decimal places, then prepared and analyzed as per the MGO standards.

### Commercial MGO Standard Analysis

The AccuStandard MGO standard (0, 100, 200, 300, 400 μL) were added by pipette to four tared glass tubes with approximately 300 mg of the honey used previously. The masses of honey and standard were recorded to four decimal places. These were mixed with 2.0 mL derivatizing reagent and left for 75 minutes before analysis by HPLC.

Two Sigma Aldrich MGO ~40% w/w solutions were diluted to nominal 1000 ppm concentrations (500 mg 40% w/w MGO solution/200.0 mL water). Duplicate samples (250 μL) were taken and assayed in separate tared test tubes, with the corresponding mass of each aliquot recorded to four decimal places before derivatization and analysis by HPLC. The second commercial standard labelled 1003 ppm MGO was also prepared by the same method as duplicate 300 μL samples.

### *Leptospermum* Honey Sample Preparation

Triplicate 300–400 mg subsamples of each of the stirred *Leptospermum* honey samples (n = 6) were added to tared glass tubes and the masses recorded to four decimal places. These honeys were derivatized with 2.0 mL of PFBHA and assayed by HPLC.

### Chromatographic Conditions

Chromatographic analysis was conducted with a Perkin Elmer FX HPLC and Autosampler, dual FX10LC Pumps and Flexar FX UV/VIS Detector with a Synergi 4μFusion-RP 80A column (75 mm × 4.6 mm, 4 μm particle size). Flow rate was 1.2 mL/min and sample injection volume was 20 μL. Mobile Phase A (MPA) was water: ACN 75/25 (v/v), and Mobile Phase B (MPB) was 100% ACN. The gradient program was 100% MPA (3.0 min) to grading to 10% MPA (11–12.5 min), then returning to 100% MPA (12.8–13.5 min). This concentration was isocratic for 0.7 min and peak detection was recorded at 263 nm.

### Data Collection and Analysis

**Fig 1:**
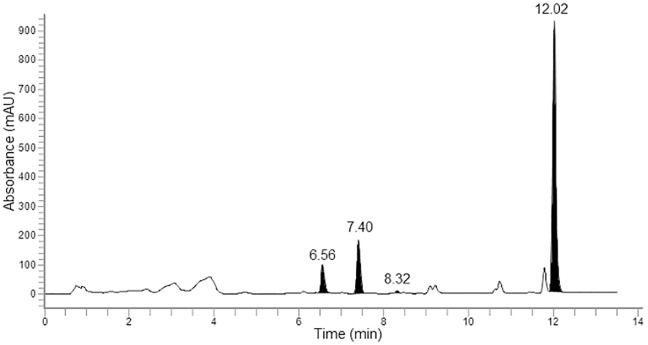
HPLC chromatogram at λ = 263 nm of a derivatized honey sample displaying peaks of interest. Dihydroxyacetone (6.56 min), anisole internal standard (7.40 min), 5-hydroxymethylfurfural (8.32 min) and methylglyoxal (12.02 min).

Calibration curves for quantitation were obtained by calculating the ratio of the peak area of the compound of interest to the peak area of the anisole internal standard in the chromatograph (Fig 1). These were then plotted against the mass of MGO, DHA and HMF in each standard.

The mass of each analyte in the sample honeys was determined by a linear regression analysis. Subsequent division of analyte mass (μg) by honey mass (g) provided concentrations for DHA, MGO and HMF in μg/g (ppm). This quantitation process was then completed for the commercial MGO standards and solutions.

Descriptive statistics and Relative Standard Deviation (% RSD) were calculated using Microsoft Excel (v16).

## Results and Discussion

**Fig 2:**
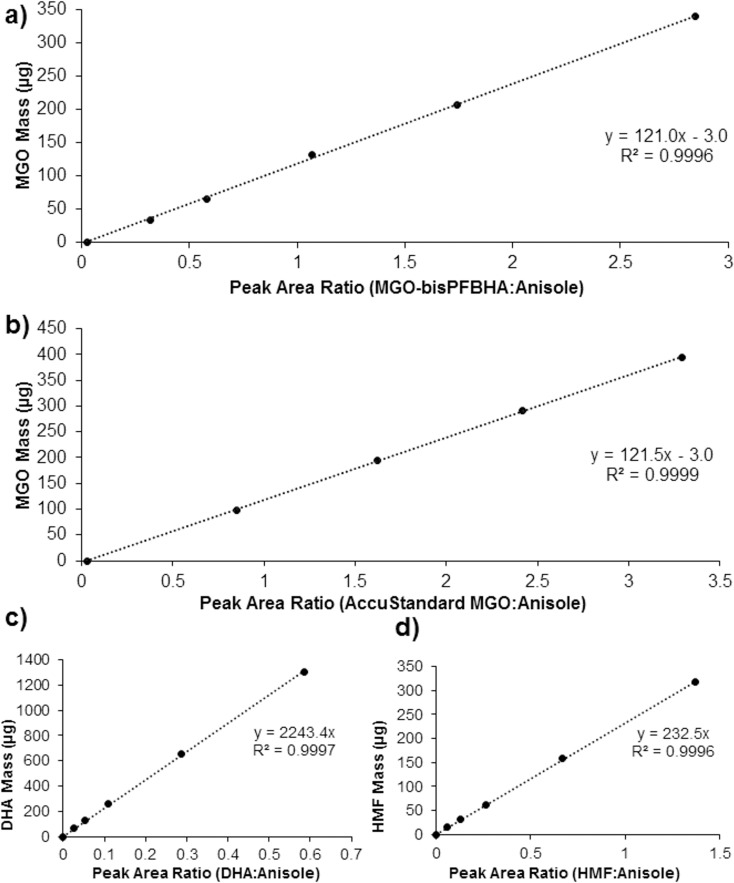
Quantitation graphs used to calculate mass (μg) of analyte in each calibration standard. Mass plotted against ratio of analyte peak area to anisole internal standard peak area at λ = 263 nm. (a) Methylglyoxal-bisPFBHA derivative. (b) AccuStandard grade methylglyoxal. (c) Dihydroxyacetone. (d) 5-Hydroxymethylfurfural.

The Anisole internal standard presented here has improved performance and reliability in comparison with currently published techniques. Combining sample dilution with a stable internal standard has improved the productivity of the method without sacrificing accuracy or precision of quantitation, as calibration curves derived for quantitation were linear for the compounds of interest (R^2^ = 0.999, Fig 2). Importantly, there is a strong correlation between the MGO linear calibrations for the recrystallized MGO-bisPFBHA standard (Fig 2a) and the commercial AccuStandard MGO (Fig 2b). This demonstrates that the MGO-bisPFBHA derivative is a reliable calibration standard comparable with the commercial AccuStandard product, which can be readily prepared in the laboratory

The equations derived from the linear regression analysis of these calibrations were used to quantitate the concentrations of DHA, MGO and HMF in six *Leptospermum* honeys [4]. There was a low mean %RSD for these data, suggesting there was precise quantitation for the analytes of interest (Table 1).

**Table 1:** Methylglyoxal, dihydroxyacetone and 5-hydroxymethylfurufal concentrations for *Leptospermum* honeys.

Sample	Dihydroxyacetone			5-Hydroxymethylfurfural			Methylglyoxal		
	Mean ± Range (mg/kg)	SD	%RSD	Mean ± Range (mg/kg)	SD	%RSD	Mean ± Range (mg/kg)	SD	%RSD
1	2290 ± 93	47	2	not detectable	<1	<1	62 ± 1	<1	<1
2	3477 ± 22	11	<1	34 ± 6	3	10	1593 ± 12	6	<1
3	240± 18	9	4	83 ± 5	2	3	290 ± 15	8	3
4	374 ± 43	23	6	12 ± 1	<1	<1	84 ± 6	4	4
5	2108 ± 37	19	1	38 ± 1	<1	<1	1004 ± 4	2	<1
6	53 ± 8	4	8	7 ± 0	<1	<1	4 ± 2	1	24

SD, standard deviation; %RSD, % relative standard deviation.

Samples tested in triplicate.

Precise quantitation of minimum MGO is useful, as MGO in honey often does not display bactericidal concentration during *in vitro* analysis until it reaches 150–200 mg/kg [2, 12]. The Anisole method has an apparent minimum effective quantitation limit for MGO at 10 ppm, as evidenced by the high %RSD for the sample with lowest MGO content (Table 1). Further testing of this method may further resolve a minimum detection limit, for example a minimum of 20 ppm has been reported elsewhere [4]. Reliable minimum detection minimums for HMF and DHA could also be resolved as part of further analyses.

The MGO standard solutions were prepared separate to the dual DHA and HMF standard as the MGO derivative is insoluble in water, while latter compounds are readily soluble in this solvent. Furthermore, honey typically contains a small interference equating to 3 ppm of MGO at the elution time of the MGO-bisPFBHA derivative. Therefore, the MGO standards were prepared with a blank honey that has been previously assayed to account for this interference and improve the precision of quantitation. The MGO mass within the recrystallized MGO-bisPFBHA standard solutions were determined by equation.

MassMGO(μg)=[MGO−bisPFBHA]×mass aliquotACN density×MWMGOMWMGO−bisPFBHA (1)

Simplified, the concentration of the MGO-bisPFBHA standard was 4450 μg/mL, which is multiplied by the mass of standard solution added (accurate to four decimal places) and then divided by the density of acetonitrile (0.786 g/mL). This provides a more reliable measure of volume than the nominal autopipette settings. Finally, the MGO in the recrystallized derivative is 0.155902 as determined by mass ratio. This results in the calibration equation for the recrystallized standard.

MassMGO(μg)=4450×mass aliquot0.786×0.155902 (2)

The commercial AccuStandard MGO (1001 ppm) yielded a linear correlation that was almost identical to the MGO-bisPFBHA derivative (Fig 2a and 2b), showing that both are suitable for use as calibration standards. The equation for calculating the mass of MGO using the AccuStandard can be found using the labelled concentration.

MassMGO(μg)=mass aliquot0.786×1001 (3)

Analysis of the two Sigma Aldrich MGO solutions and an unnamed MGO standard were assessed by comparing their described concentrations against their MGO content as determined by the linear equation derived from the recrystallized MGO-bisPFBHA standard (Table 2).

**Table 2:** Comparison between theoretical concentration of commercial methylglyoxal solutions and the results of quantitation method using the anisole technique.

Sample	Labelled Concentration	Determined concentration
Sigma Aldrich 1	38.7% w/w	35.1% w/w
Sigma Aldrich 2	40.0% w/w	41.3% w/w
Standard *(name withheld)*	1003 ppm	719 ppm

Samples tested in duplicate.

The described concentrations for MGO in these solutions were inconsistent when compared to the quantitation from the MGO-bisPFBHA derivative (Table 2). For example, the unnamed commercial MGO standard was found to be 285 ppm different from the concentration on the label (1003 ppm). The two MGO solutions from Sigma were found to be 3.6% and 1.3% different from their described values at date of manufacture. These discrepancies may arise from decomposition due to aging or varying storage conditions during transit, however all materials were kept in accordance with storage conditions and tested within their expiry dates.

Precise and timely MGO quantitation is vital for honey research and industry. The results suggest calibrations which utilize some commercial MGO solutions and standards as their quantitation standard may have unreliable MGO quantitation. The recrystallized MGO-bisPFBHA derivative is easily produced in the laboratory and can be prepared to a precise concentration. Alternatively, the AccuStandard MGO standard was also found to provide faithful quantitation. Therefore, using this commercial standard or preparing the derivative for quantitation would avoid calibration bias.

Previous PFBHA methods have used a small mass of HA as the internal standard and this required derivatization for quantitation. This standard solution was found to be unstable at room temperature and was prepared in bulk before aliquots were frozen for single use. In contrast, anisole is a stable compound that produces a consistent and discrete peak in the chromatograph without requiring derivatization (Fig 1). This retains the sensitivity of the previous internal standard while also reducing waste as the anisole internal standard in the sample diluent remains stable over for long term use when refrigerated (4°C) and is therefore readily available for immediate use.

The PFBHA method involved sample dilution by adding water and ACN to the derivatized honey solutions until they were visibly homogeneous. This is time inefficient and subjective. The Anisole method combines the internal standard with a dilution solvent comprised of water and ACN in a ratio that our laboratory found was commonly required to achieve complete dilution of derivatized compounds for most honeys, although extra dilution is still required for samples with exceptionally high MGO content. Furthermore, under the HPLC conditions described, all compounds are fully resolved in 13.5 minutes, without sacrificing efficacy. Consequently, our protocol can produce accurate data more efficiently than previously described [4].

The new Anisole method described here offers precise and efficient quantitation of the compounds of interest in *Leptospermum spp*. honey. This is accomplished by complete derivatization of MGO, DHA and HMF followed by careful addition of a known volume of sample diluent spiked with anisole as an internal standard immediately before chromatographic assay. This reduces the risk of an unstable internal standard and improves the efficiency of the task by simultaneously dissolving the derivatized samples. Our recommendation for using recrystallized MGO-bisPFBHA derivative or using the AccuStandard brand MGO as a quantitation standard eliminates bias that may arise if some commercial MGO solutions are used as the quantitation standard. Using either of these products as the MGO standard contributes to the reliability and accuracy of the Anisole method by producing a reliable quantitation standard for this important compound in *Leptospermum spp*. honey.

## Supporting Information

S1 Table Dihydroxyacetone (DHA) and 5-hydroxymethylfurfural (HMF) dual standard. PA: peak area at λ = 263 nm.
(XLSX)

S2 Table AccuStandards Methylglyoxal (MGO) standard. PA: peak area at λ = 263 nm.
(XLSX)

S3 Table Methylglyoxal-bis *o*-(2,3,4,5,6-Pentafluorobenzyl) hydroxylamine (MGO-bisPFBHA) derivative standard. PA: peak area at λ = 263 nm.
(XLSX)

S4 Table Methylglyoxal (MGO), dihydroxyacetone (DHA) and 5-hydroxymethylfurfural (HMF) content in selected honeys (n = 6). PA: peak area at λ = 263 nm; PPM: parts per million.
(XLSX)

S5 Table Quantitation of commercial methylglyoxal (MGO) solutions. PA: peak area at λ = 263 nm; PPM: parts per million.
(XLSX)

## Abbreviations

ACN: acetonitrile
DHA: dihydroxyacetone
HMF: 5-hydroxymethylfurfural
HPLC: High Performance Liquid Chromatography
MGO: methylglyoxal
NPA: non-peroxide antibacterial activity
PFBHA: *o*-(2,3,4,5,6-Pentafluorobenzyl) hydrochloride
